# Diagnostic value of PRIMARY score and a combined model with SUVmax in 18F-PSMA-1007 PET/CT for prostate cancer

**DOI:** 10.3389/fmed.2026.1829352

**Published:** 2026-05-11

**Authors:** Futian Hu, Xiangru Li, Zonglin Li, Weiqiang Huang, Wenjie Liu, Shidong Lian, Shiquan Liu

**Affiliations:** The Second Affiliated Hospital of Guangxi Medical University, Nanning, China

**Keywords:** clinically significant prostate cancer, positron emission tomography computed tomography, PRIMARY score, prostate-specific membrane antigen, prostatic neoplasms, standardized uptake value

## Abstract

**Objective:**

To investigate the diagnostic value of the PRIMARY score in 18F-PSMA-1007 PET/CT for prostate cancer (PCa), to compare its diagnostic efficacy with maximum standardized uptake value (SUVmax), and explore the value of their combined model.

**Methods:**

A total of 180 patients with suspected prostate cancer were initially screened. After excluding 91 patients (70 with prior treatment, 16 without pathological confirmation, 3 with unclear pathological results, and 2 with poor image quality), 89 patients were finally enrolled. We retrospectively reviewed 89 patients with suspected PCa who underwent 18F-PSMA-1007 PET/CT from November 2023 to September 2025, including 61 with PCa and 28 with benign lesions. All patients underwent standard preoperative evaluation including PSA, digital rectal examination (DRE), ultrasound, or multiparametric MRI (mpMRI) before imaging. Prostate biopsy was performed within 2 weeks before or after PET/CT according to clinical indications. The SUVmax of the representative lesion was measured and the PRIMARY score was recorded. Intergroup differences were analyzed, optimal cutoff values were determined by ROC curve, and AUCs were compared by the DeLong test. Risk stratification was performed for clinically significant prostate cancer (csPCa), with focus on the combined diagnostic strategy for “gray-zone” lesions with PRIMARY score 4. Interobserver agreement was evaluated using the Kappa coefficient.

**Results:**

Age was comparable between the two groups (*P* > 0.05), while SUVmax and PRIMARY score were significantly different (both *P* < 0.05). The PRIMARY score showed excellent interobserver agreement (Kappa = 0.903). For PCa diagnosis, the AUCs of SUVmax, PRIMARY score, and the combined index PRE were 0.921, 0.960, and 0.963, and PRE was significantly superior to SUVmax alone, but not significantly different from PRIMARY score. For csPCa, the positive predictive value was 97.6% for PRIMARY score 5, 68.2% for score 4, and increased to 85.7% by applying the combined criterion (PRIMARY = 4 and SUVmax ≥ 7.5). Notably, 3 of 8 patients in the PRIMARY = 4 and SUVmax < 7.5 subgroup still had csPCa, indicating a trade–off between PPV and missed csPCa. PRIMARY score ≤ 3 showed a low csPCa prevalence of 7.7%.

**Conclusion:**

In 18F-PSMA-1007 PET/CT, the PRIMARY score demonstrates comparable diagnostic efficacy to SUVmax with excellent reproducibility. The combined PRE model was superior to SUVmax alone, but not significantly different from PRIMARY score in this cohort, optimizing the stratification of PRIMARY score 4 “gray-zone” lesions. This approach effectively reduces false positives and provides a robust quantitative reference for biopsy decisions and individualized patient management.

## 1 Introduction

Prostate cancer (PCa) is the second most common malignant tumor in men worldwide, and its incidence continues to rise as the population ages (1). The accurate diagnosis of PCa has become a major focus of clinical concern. Traditional screening methods, such as prostate-specific antigen (PSA), ultrasound, and multiparametric MRI (mpMRI), have certain limitations and struggle to meet clinical demands. Studies have shown that, compared with conventional imaging, prostate-specific membrane antigen (PSMA) PET/CT can identify lesions more accurately and lead to clinical upstaging in approximately 20% of PCa patients (2, 3).

Currently, the most easy to obtain and straightforward among semi-quantitative parameters in PSMA PET/CT, although not validated for routine use with this radiopharmaceutical, is the maximum standardized uptake value (SUVmax). However, SUVmax is a semi-quantitative parameter susceptible to various confounding factors. This results in variations in diagnostic thresholds across different PET/CT centers, and a unified, reproducible standard has yet to be established (4–6). To address this issue, Emmett et al. first proposed the standardized, visual-based PRIMARY scoring system in 2022, which integrates prostatic anatomical zones with lesion uptake characteristics (7). Nevertheless, this scoring system was originally developed using 68Ga-labeled PSMA tracers, and its applicability to the increasingly widespread ^18^F-labeled PSMA tracers remains to be validated.

Therefore, this study aims to verify the diagnostic value of the PRIMARY scoring system in ^18^F-PSMA-1007 PET/CT, compare its diagnostic performance with SUVmax, explore the value of their combined application, and propose an interpretation strategy for “gray-zone” lesions.

## 2 Materials and methods

### 2.1 Study population

**FIGURE 1:**
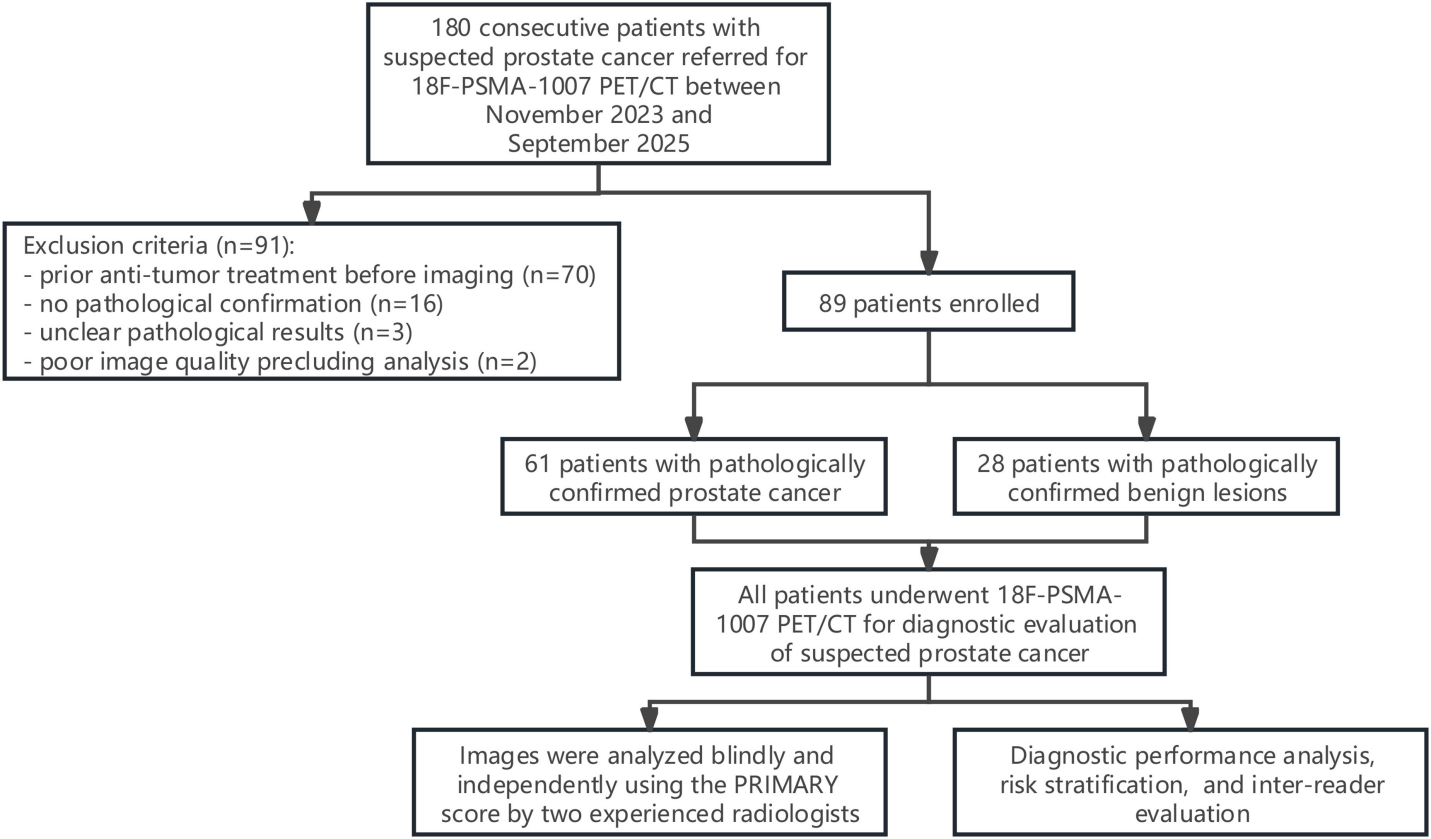
Study flowchart showing patient inclusion and exclusion according to STARD 2015.

This was a single-center retrospective study. Patients with suspected PCa who underwent 18F-PSMA-1007 PET/CT at the Department of Urology of our hospital between November 15, 2023 and September 23, 2025 were enrolled (Figure 1). All patients were referred for PET/CT due to elevated PSA (>4.0 ng/ml or progressive increase) and/or abnormal DRE, ultrasound findings suggestive of suspicious malignant nodules, or mpMRI findings for initial diagnostic evaluation.

#### 2.1.1 Inclusion criteria

1. All patients had pathological confirmation via transrectal or transperineal ultrasound-guided systematic 12-core biopsy or radical prostatectomy specimen. Benign lesions were confirmed by negative systematic biopsy, and all benign patients were followed up for at least 3 months with PSA and prostate ultrasound; mpMRI or other imaging reviews were performed when necessary. No progressive elevation in PSA or new abnormal imaging findings was observed during follow-up.
2. PET/CT examination and pathological diagnosis were completed within 1 month.

#### 2.1.2 Exclusion criteria

1. Previous surgery, endocrine therapy, or other antineoplastic treatments before imaging;
2. Poor image quality precluding analysis;
3. Unclear clinical or pathological results.

This study was approved by the Ethics Committee, and written informed consent was waived for this retrospective study.

### 2.2 Data collection and grouping

A total of 180 patients were initially screened, and 91 were excluded. Finally, 89 patients were enrolled, aged 58–87 years, with a mean age of 71.8 ± 6.8 years (mean ± SD). Patients were divided into two groups according to pathological results:

- Prostate cancer (PCa) group (*n* = 61): all adenocarcinomas, including 57 cases of clinically significant prostate cancer (csPCa, Gleason score ≥ 7) and 4 cases of non-clinically significant prostate cancer (non-csPCa, Gleason score < 7).
- Non-prostate cancer (non-PCa) group (*n* = 28): benign prostatic hyperplasia with chronic inflammation (*n* = 15), simple benign prostatic hyperplasia (*n* = 9), normal prostate tissue (*n* = 3), and benign prostatic hyperplasia with local suppurative inflammation (*n* = 1).

Pathological modality: 53 patients underwent systematic 12-core biopsy, and 36 patients underwent radical prostatectomy. All benign cases had negative biopsy results.

### 2.3 Study methods

#### 2.3.1 18 F-PSMA-1007 PET/CT acquisition protocol

18F-PSMA-1007 was injected intravenously at a dose of 3.70–5.55 MBq/kg (radiochemical purity > 99%). Whole-body PET/CT scanning was performed using a Siemens Biograph mCT scanner at (60 ± 10) min after injection, from the top of the skull to the middle of the thighs.

CT parameters: tube voltage 120 kV, tube current 320 mAs for the head and 140 mAs for the body, slice thickness 3 mm. PET scans were acquired in 3D mode, and PET images were obtained by iterative reconstruction (OSEM algorithm) with parameters set as follows: 4 iterations and 16 subsets. CT data were used for PET attenuation correction and anatomical localization.

#### 2.3.2 Image analysis

The lesion with the highest PRIMARY score was selected as the target lesion. If the PRIMARY scores were equal, the lesion with the highest SUVmax was chosen as the representative lesion for analysis.

Two radiologists with more than 3 years of experience in PET/CT diagnosis independently interpreted images in a blinded manner. Discrepancies were resolved by consensus. Image fusion and quantitative analysis were performed on a Navigator workstation. For lesions with abnormally increased radioactive uptake in the prostate, a volume of interest (VOI) was delineated using the standard threshold algorithm built into the equipment, and the maximum standardized uptake value (SUVmax) of the lesion was automatically calculated.

A 1.0 cm^2^ circular region of interest (ROI) was drawn in normal prostate tissue adjacent to the lesion as background. The lesion-to-background ratio (LBR) was calculated to assist in assigning the PRIMARY score.

#### 2.3.3 PRIMARY scoring criteria and interobserver agreement

- Score 1: Absence of the following patterns;
- Score 2: Non-focal high uptake in the transition zone or central zone;
- Score 3: Focal high uptake in the transition zone with LBR ≥ 2;
- Score 4: Focal high uptake in the peripheral zone;
- Score 5: SUVmax ≥ 12.0 in any zone.

Two readers independently assigned PRIMARY scores. Interobserver agreement was evaluated using the Kappa value. Kappa ≥ 0.81 was considered almost perfect agreement.

#### 2.3.4 Development of the combined diagnostic index (PRE)

To evaluate the potential benefit of combining the PRIMARY score and SUVmax, an exploratory combined analysis was performed. Binary logistic regression was conducted with pathological outcome (PCa = 1, non-PCa = 0) as the dependent variable, and SUVmax and PRIMARY score (both as continuous variables) as independent variables. The PRIMARY score is an ordinal variable, but was treated as continuous for exploratory modeling purposes.

Multicollinearity was tested using the variance inflation factor (VIF). VIF < 5 indicated acceptable collinearity and supported the feasibility of combined analysis.

The regression equation was: logit(P)​ =​ −7.31​ +​ 1.85 × PRIMARY + 0.15 × SUVmax

The probability value P output by the regression equation was defined as the exploratory combined diagnostic index PRE (range 0–1).

### 2.4 Statistical analysis

Statistical analysis was performed using SPSS 27.0 software. The Shapiro–Wilk test was used to assess normality of measurement data. Normally distributed data were expressed as mean ± SD and compared using independent-samples *t*-test. Non-normally distributed data were expressed as median (interquartile range, P25, P75) and compared using the Mann–Whitney U test.

Kappa analysis was used to evaluate interobserver agreement for the PRIMARY scores between the two readers. ROC curves were generated for SUVmax, PRIMARY score, and PRE to calculate the area under the curve (AUC), optimal cut-off value, sensitivity, specificity, and Youden index (YI). AUCs were compared using the DeLong test. A two-sided test was used with α = 0.05, and *P* < 0.05 was considered statistically significant.

## 3 Results

### 3.1 Comparison of variables between groups

Age did not differ significantly between the two groups (*P* > 0.05). SUVmax and PRIMARY score were significantly different between the PCa and non-PCa groups, and between the csPCa and other groups (all *P* < 0.001) (Tables 1, 2).

**TABLE 1:** Comparison of baseline characteristics between PCa and non-PCa groups.

Variables	PCa (*n* = 61)	Non-PCa (*n* = 28)	Statistic (*t/Z*)	*P*-value
Age (years)	72.4 ± 6.9	70.6 ± 6.5	1.109	0.27
PRIMARY score	5 (4, 5)	2 (2, 3)	−7.391	<0.001
SUVmax	16.5 (11.0, 26.4)	5.2 (4.5, 6.2)	−6.349	<0.001

**TABLE 2:** Comparison of baseline characteristics between cs-PCA and other groups.

Variables	cs-PCa (*n* = 57)	Other (*n* = 32)	Statistic (*t/Z*)	*P*-value
Age (years)	72.1 ± 6.8	71.3 ± 6.9	−0.526	0.600
PRIMARY score	5 (4, 5)	2 (2, 3)	−7.139	<0.001
SUVmax	17.7 (11.7, 26.9)	5.2 (4.5, 6.2)	−6.588	<0.001

**FIGURE 2:**
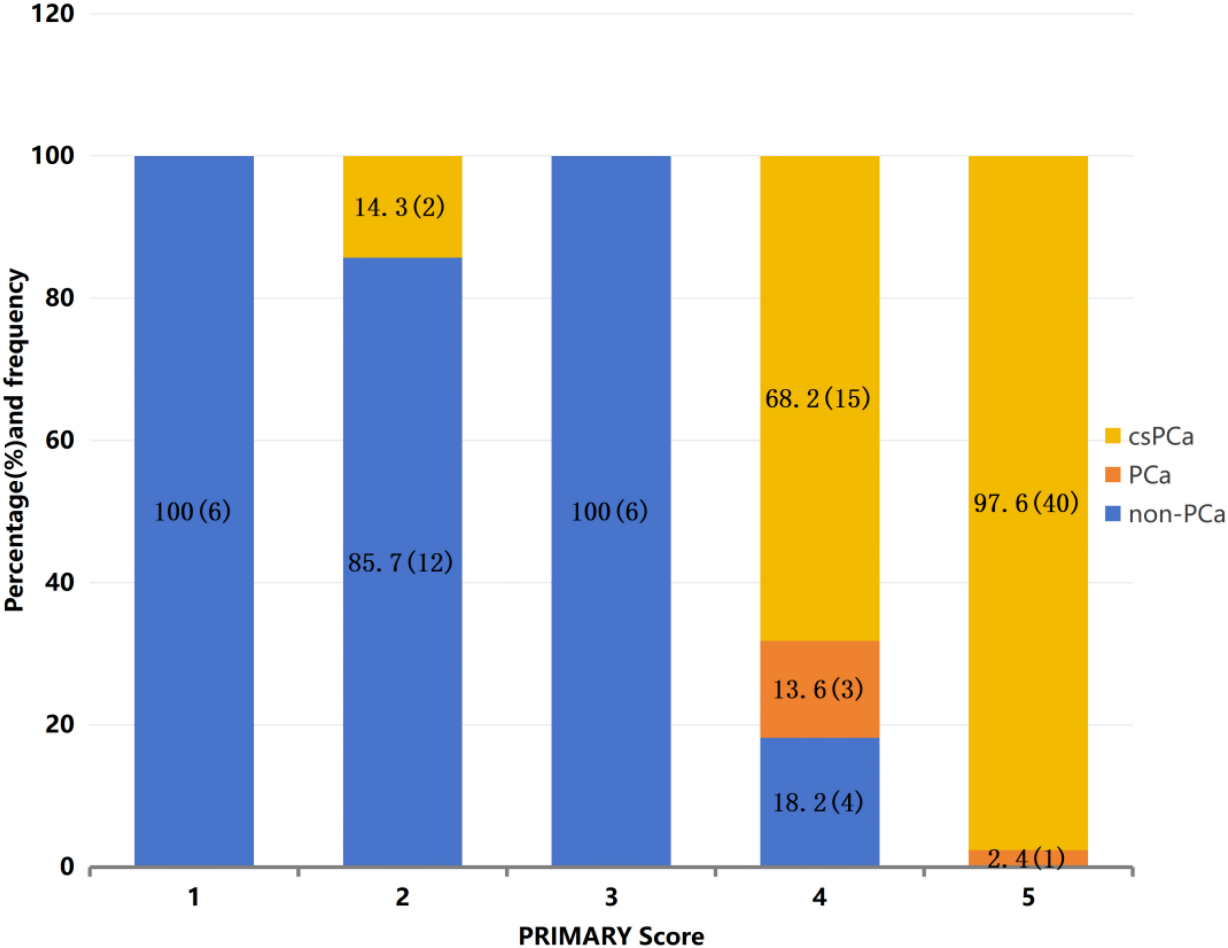
Detailed distribution of PRIMARY scores among the patients.

Interobserver agreement analysis showed good consistency between the two readers for the PRIMARY score (Kappa = 0.903). Among 89 patients, the distribution of PRIMARY scores was as follows: score 1 (*n* = 6), score 2 (*n* = 14), score 3 (*n* = 6), score 4 (*n* = 22), score 5 (*n* = 41). The PCa group was dominated by scores 4–5 (59/61), while the non-PCa group was dominated by scores 1–3 (24/28) (Figure 2).

### 3.2 Diagnostic performance of each index

ROC analysis was performed for SUVmax and PRIMARY score, both of which showed significant intergroup differences. Multivariate logistic regression analysis showed that the PRIMARY score contributed strongly to the prediction of prostate cancer (β = 1.85), and SUVmax was also included in the model as a quantitative supplement (β = 0.15).

**FIGURE 3:**
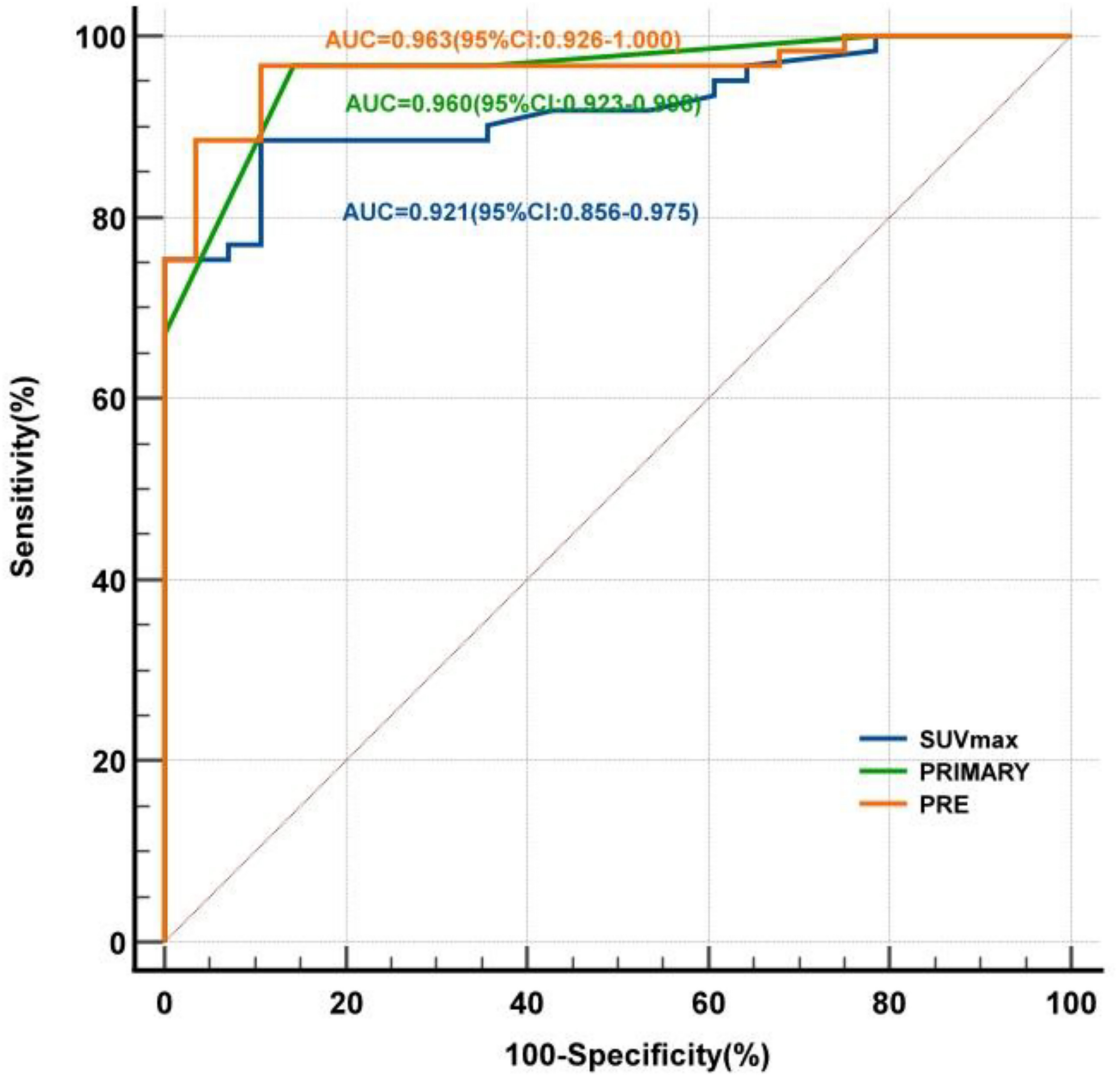
ROC curves for SUVmax, PRIMARY score, and PRE.

ROC curves of each index were subsequently plotted (Figure 3). In this study cohort:

- SUVmax for diagnosing prostate cancer: optimal cut-off value 7.5, sensitivity 88.5%, specificity 89.3%, YI 0.778, AUC 0.921 (95% CI: 0.866–0.975);
- PRIMARY score for diagnosing prostate cancer: optimal cut-off value 4, sensitivity 96.7%, specificity 85.7%, YI 0.824, AUC 0.960 (95% CI: 0.923–0.998);
- Combined diagnostic index PRE for diagnosing prostate cancer: optimal cut-off value 0.692, sensitivity 96.7%, specificity 89.3%, YI 0.860, AUC 0.963 (95% CI: 0.926–1.000).

DeLong test showed that the AUC of PRE was significantly different from that of SUVmax (*Z* = −2.309, *P* = 0.021). No significant differences were found between PRE and PRIMARY score, or between PRIMARY score and SUVmax (all *P* > 0.05) (Table 3).

**TABLE 3:** Comparison of AUCs.

Paired indicators	*Z*-value	*P*-value
PRIMARY score vs. SUVmax	1.796	0.072
PRIMARY score vs. PRE	−0.344	0.731
SUVmax vs. PRE	−2.309	0.021

### 3.3 Internal assessment of the exploratory combined index PRE

Internal validation of the combined index PRE was performed using the Bootstrap method (1000 resamples), and the Hosmer–Lemeshow (H–L) goodness-of-fit test was performed.

**FIGURE 4:**
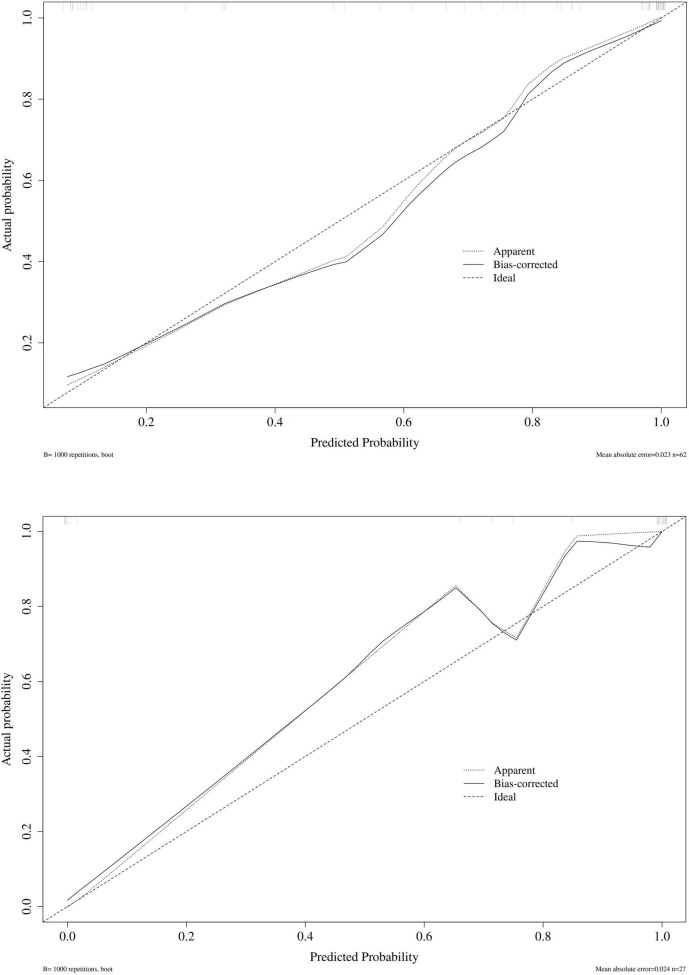
Calibration curves for the training set and validation set.

Results showed that in the original fitting group, H–L test χ^2^ = 4.461, df = 8, *P* = 0.813; in the Bootstrap validation group, χ^2^ = 0.788, df = 8, *P* = 0.999. Both *P* > 0.05, indicating good consistency between the predicted probability of PRE and the actual incidence (Figure 4).

**FIGURE 5:**
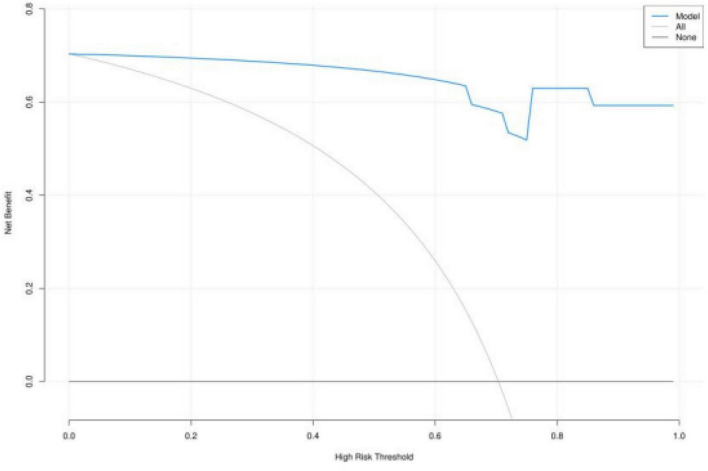
Decision curve analysis of the combined model PRE.

Decision curve analysis showed that within the threshold probability range of 0–1, the net benefit of the PRE model for diagnosing PCa was higher than that of the “all biopsy” (All) and “no biopsy” (None) strategies, especially within the range of 0–0.65 (Figure 5).

### 3.4 Risk stratification for csPCa and “gray-zone” strategy

Patients were divided into a csPCa group and an other group (including non-csPCa and non-PCa) with csPCa as the main study endpoint. PRIMARY score and SUVmax were significantly different between the two groups (all *P* < 0.001).

Stratification results were as follows:

- PRIMARY score 5: positive predictive value (PPV) for csPCa was 97.6% (40/41);
- PRIMARY score 4: defined as a “diagnostic gray zone” in this study due to its moderate PPV (68.2%). After applying the combined criterion “PRIMARY = 4 and SUVmax ≥ 7.5” in this gray-zone subgroup, the PPV increased to 85.7% (12/14). However, 3 of 8 patients in the PRIMARY = 4 and SUVmax < 7.5 subgroup still had csPCa, indicating that this threshold improves PPV but may miss a considerable proportion of csPCa if biopsy is deferred.
- PRIMARY score ≤ 3: prevalence of csPCa was only 7.7% (2/26).

## 4 Discussion

This study compared the diagnostic performance of the PRIMARY score, SUVmax, and the exploratory combined index PRE for prostate cancer, and performed an internal evaluation of PRE based on calibration and clinical net benefit. The results showed that PRE possesses favorable discrimination and calibration consistency in this cohort, suggesting its clinical application value.

The accurate diagnosis of prostate cancer is the cornerstone of individualized treatment. Current clinical practice faces two core tasks: first, to accurately determine whether a lesion is prostate cancer (PCa); and second, to further identify clinically significant prostate cancer (csPCa) that requires active intervention. This study confirms that both the PRIMARY score and the traditional quantitative indicator SUVmax are of great value in diagnosing prostate cancer. The combined strategy constructed by integrating the advantages of both can optimize the judgment of “gray-zone” lesions, which have ambiguous risk assessment and difficult clinical decision-making under a single evaluation system; it improves the positive predictive value (PPV) of csPCa and provides a reference for addressing the interpretation dilemma of “gray-zone” lesions in PSMA-PET/CT diagnosis.

The PRIMARY score is constructed based on prostatic anatomical zones (peripheral zone, transition zone, and central zone) and lesion uptake characteristics (focal/non-focal high uptake, LBR, SUVmax), possessing standardization and reproducibility (7, 8). Previous studies have shown that the diagnostic AUC of this score in 68Ga-PSMA PET/CT imaging is 0.85–0.91, and in 18F-PSMA-1007 PET/CT imaging is 0.91–0.955; the values corresponding to 18F-labeled ligands are slightly higher, but the difference is not statistically significant (7, 9). Our findings are consistent with previous reports, with the AUC of the PRIMARY score for diagnosing prostate cancer being 0.960, which showed no statistically significant difference (*P* = 0.072) compared to the traditional indicator SUVmax (AUC = 0.921). This result suggests that the PRIMARY score showed favorable diagnostic performance in this 18F-PSMA-1007 cohort; direct head-to-head comparison with 68Ga-labeled tracers is needed to confirm ligand-independent universality. It can provide a standardized grading framework, improve inter-center consistency, and solve the clinical pain point of SUVmax diagnostic threshold heterogeneity. The sensitivity of the PRIMARY score (96.7%) is higher than that of SUVmax (88.5%), suggesting its advantage in the detection of suspicious lesions. This feature originates from the design logic of PRIMARY: based on the anatomical characteristics that prostate cancer commonly occurs in the peripheral zone and benign lesions mostly originate in the transition zone, assigning a higher diagnostic weight to focal high uptake in the peripheral zone (PRIMARY score 4) helps in the early identification of lesions.

Previous studies have shown that the PRIMARY score combined with multiparametric MRI and PSA-related indicators can significantly enhance the diagnostic performance for prostate cancer (AUC 0.85–0.978) (9, 10). The results of this study showed that the diagnostic specificity of the PRIMARY score was 85.7%, which was lower than the 89.3% of SUVmax. This difference suggests that the diagnostic performances of the two possess their own respective advantages and may be complementary. Given that a correlation may exist between the two, we performed a multicollinearity test, and the results showed that the variance inflation factor (VIF = 1.63) was far below the critical value (such as 5 or 10); thus, the influence of multicollinearity is negligible, supporting the feasibility of the combined analysis. Notably, PRIMARY score 5 is defined by SUVmax ≥ 12.0, leading to partial overlap in biological signal between PRIMARY and SUVmax. Although low VIF excludes significant statistical collinearity, the conceptual overlap should be acknowledged, and the PRE model remains exploratory.

**FIGURE 6:**
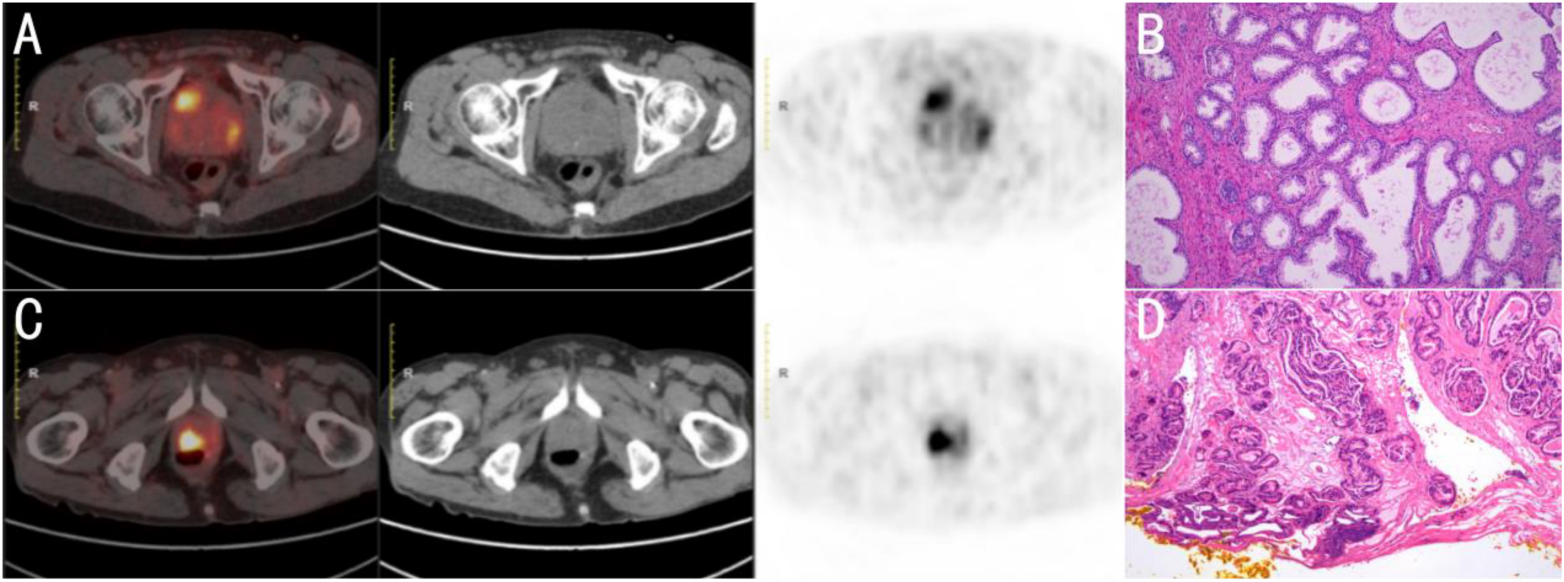
Positron emission tomography/computed tomography (PET/CT) imaging and postoperative histopathological results of the patients. In Patient A **(A,B)**, focal PSMA uptake was noted in the right peripheral zone of the prostate, assigned a PRIMARY score of 4 and SUVmax of 7.3; histopathology after surgery demonstrated benign prostatic hyperplasia. In Patient B **(C,D)**, focal PSMA uptake was identified at the prostatic apex, with a PRIMARY score of 4 and SUVmax of 8.9; surgical pathology confirmed adenocarcinoma.

The combined index PRE constructed based on this achieved the optimal comprehensive diagnostic performance, with an AUC of 0.963, which is similar to the diagnostic performance of PRIMARY combined with other indicators in previous studies. Compared with single indicators, the overall AUC of PRE was comparable to that of PRIMARY and significantly superior to SUVmax (ΔAUC = 0.042, *P* = 0.021). The sensitivity of PRE was 96.7%, the specificity was 89.3%, and the Youden index was 0.860. By integrating the high sensitivity of the PRIMARY score with the high specificity of SUVmax, PRE compensates for the limitations of single indicators and optimizes diagnostic performance, particularly showing prominent performance in solving the “gray-zone” problem and reducing the false-positive rate. Among the 22 lesions with a PRIMARY score of 4, benign lesions accounted for 18.2% (4/22). The study by Li et al. (11) confirmed that such false-positive lesions are mostly related to prostate atrophy with cyst formation, adenomatous hyperplasia, or inflammation, and their median SUVmax (7.15) was significantly lower than that of true-positive lesions (16.64). Based on this pattern, this study exploratorily set a combined judgment criterion of “PRIMARY = 4 and SUVmax ≥ 7.5.” After application, the proportion of non-prostate cancer in this subgroup decreased to 7.1% (1/14) (Figure 6). This combined criterion improves PPV but does not support routine biopsy omission, as 3 of 8 patients with SUVmax < 7.5 still had csPCa. Shared decision-making rather than pure biopsy-sparing is recommended in this subgroup. This study optimizes the solution for “gray-zone” problems through a combined diagnostic strategy, echoing the integrated PSMA PET/CT reporting concept promoted by the SPARC consensus (12).

Regarding the imbalance between csPCa and non-csPCa in our cohort, we acknowledge the high prevalence of csPCa (57/61, 93%) among malignant lesions, which reflects the real-world distribution of patients referred for initial diagnostic PSMA PET/CT at our center. Importantly, the added value of this combined strategy is not to increase the overall proportion of csPCa, but to improve risk stratification within the ambiguous PRIMARY = 4 “gray zone.” In our cohort, the prevalence of csPCa in PRIMARY = 4 lesions was 68.2% (15/22) without SUVmax stratification. After applying the SUVmax ≥ 7.5 threshold, the positive predictive value (PPV) for csPCa increased to 85.7% (12/14) in the high-risk subgroup, while the remaining 8 patients with SUVmax < 7.5 had a csPCa rate of only 37.5% (3/8). This allows us to better differentiate between patients at very high risk (nearly 100% csPCa probability) and those with substantially lower risk, which has clear clinical utility for guiding biopsy decisions and shared decision-making.

This study evaluated the clinical net benefit of PRE, achieving a progressive analysis from identifying PCa to screening for csPCa, which aligns with the logic of clinical precision diagnosis and treatment. Decision curve analysis suggested that PRE is helpful in identifying high-risk populations and may reduce unnecessary biopsies in this cohort; the combined judgment strategy for the PRIMARY score 4 “gray zone” focuses on the core goal of csPCa. Previous studies have suggested that both the PRIMARY score and SUVmax are positively correlated with the intensity of tumor PSMA expression, and higher PSMA expression levels are associated with stronger tumor aggressiveness; therefore, both are positively correlated with the risk of csPCa, meaning the higher the score, the greater the probability of suffering from csPCa (13–16). The results of this study are highly consistent with this, as the differences in PRIMARY scores and SUVmax between the csPCa group and other groups were statistically significant (*P* < 0.001). When the PRIMARY score was 5, the PPV for csPCa was 97.6% (40/41); when the score was 4, the PPV was 68.2% (15/22), and by secondary screening using a combined SUVmax ≥ 7.5 threshold, the PPV of this subgroup could be increased to 85.7% (12/14); while when PRIMARY ≤ 3, the prevalence of csPCa was only 7.7% (2/26), showing certain value for exclusionary diagnosis, similar to the study by Akçay et al. (17). This provides an intuitive and quantitative basis for clinical decision-making. For patients who refuse or cannot tolerate prostate biopsy, risk-stratified management can be implemented. A PRIMARY score of 5 with a PPV of 97.6% for csPCa suggests a high probability of csPCa, which can serve as an important basis for radical surgery or other treatments, although patients must be informed of an approximately 2.4% risk of overtreatment; a PRIMARY score of 4 suggests intermediate-to-high risk, and close follow-up combined with clinical indicators is recommended. If SUVmax is above the threshold, biopsy should be prioritized to confirm the diagnosis; a PRIMARY score ≤ 3 with a csPCa prevalence of only 7.7% suggests low risk, and clinical follow-up and further risk assessment (such as re-examining PSA and mpMRI every 6 months) may be considered, but patients must be informed that there remains an approximately 8% possibility of missing csPCa, consistent with the report by Shi et al. (18).

## 5 Limitations

This study has several limitations. First, this study is a single-center retrospective study with a relatively limited sample size (*n* = 89). The study population mainly consisted of patients with suspected prostate cancer who underwent biopsy, leading to a high proportion of PCa and a certain degree of selection bias, which may affect the generalizability of the conclusions. Second, the PRE index and the combined judgment criterion for the PRIMARY score 4 “gray zone” (SUVmax ≥ 7.5) proposed in this study are exploratory results constructed based on data from our center; their universality and robustness still require external validation with multi-center, large-sample data. Third, not all patients underwent radical prostatectomy, and needle biopsy involves sampling error, which may not fully reflect the true biological characteristics of the tumor. Fourth, some patients with benign findings had a relatively short follow-up period, posing a potential risk of misclassification. Finally, our study relied exclusively on PSMA PET/CT; in the future, multi-parameter models could be constructed by combining PI-RADS scores, PSA density (PSAD), and other indicators to further enhance the efficacy of identifying csPCa.

## 6 Conclusion

In conclusion, this study demonstrates that the PRIMARY score derived from ^68^Ga-PSMA PET/CT imaging is applicable to ^18^F-PSMA-1007 PET/CT, with diagnostic performance for prostate cancer comparable to SUVmax and excellent interobserver agreement, possessing the potential for application as a standardized visual grading tool. The exploratory combined diagnostic index PRE constructed based on PRIMARY and SUVmax was superior to SUVmax alone, but not significantly different from PRIMARY score in this cohort, especially for the secondary assessment of PRIMARY = 4 “gray-zone” lesions; it can reduce false positives while maintaining high sensitivity, providing a quantitative reference for biopsy decisions and individualized management.

## Data Availability

The raw data supporting the conclusions of this article will be made available by the authors, without undue reservation.
